# Imaging, biomarkers, and vascular cognitive impairment in China: Rationale and design for the VICA study

**DOI:** 10.1002/alz.14352

**Published:** 2024-11-13

**Authors:** Mei Cui, Zishuo Jin, Yingzhe Wang, Jiwei Jiang, Sisi Peng, Qiang Wei, Shuting Zhang, Qingzhang Tuo, Junchao Xie, Haixia Leng, Hongxing Wang, Yanxin Zhao, Peng Lei, Jun Xu, Kai Wang, Junjian Zhang, Yanfeng Jiang, Ding Ding, Fang Xie, Jintai Yu, Qiang Dong, VICA Investigators

**Affiliations:** ^1^ Department of Neurology Huashan Hospital Fudan University Shanghai China; ^2^ MOE Frontiers Center for Brain Science National Center for Neurological Disorders Shanghai China; ^3^ Beijing Tiantan Hospital Capital Medical University Beijing China; ^4^ China National Clinical Research Center for Neurological Diseases Beijing China; ^5^ Department of Neuropsychology Zhongnan Hospital of Wuhan University Wuhan Hubei China; ^6^ Department of Neurology the First Affiliated Hospital of Anhui Medical University Hefei Anhui China; ^7^ Department of Neurology West China Hospital Sichuan University Chengdu Sichuan China; ^8^ Department of Geriatrics and State Key Laboratory of Biotherapy National Clinical Research Center for Geriatrics West China Hospital Sichuan University Chengdu Sichuan China; ^9^ Department of Neurology Shanghai Tenth People's Hospital Tongji University School of Medicine Shanghai China; ^10^ Division of Neuropsychiatry and Psychosomatics Department of Neurology Xuanwu Hospital Capital Medical University Beijing China; ^11^ Department of Neurology and State Key Laboratory of Biotherapy West China Hospital Sichuan University Chengdu Sichuan China; ^12^ Department of Neurology Zhongnan Hospital of Wuhan University Wuhan Hubei China; ^13^ State Key Laboratory of Genetic Engineering, Human Phenome Institute, Zhangjiang Fudan International Innovation Center Fudan University Shanghai China; ^14^ Institute of Neurology, National Center for Neurological Disorders, National Clinical Research Center for Aging and Medicine, Huashan Hospital Fudan University Shanghai China; ^15^ Department of Nuclear Medicine & PET Center Huashan Hospital Fudan University Shanghai China; ^16^ Department of Neurology and National Center for Neurological Disorders Huashan Hospital, State Key Laboratory of Medical Neurobiology and MOE Frontiers Center for Brain Science, Shanghai Medical College Fudan University Shanghai China

**Keywords:** China, cohort, community, CSVD, hospital, imaging, omics research, stroke, vascular cognitive impairment

## Abstract

**INTRODUCTION:**

Vascular cognitive impairment (VCI) is highly heterogeneous, with unclear pathogenesis. Individuals with vascular risk factors (VRF), cerebral small vessel disease (CSVD), and stroke are all at risk of developing VCI. To address the growing challenges posed by VCI, the “Vascular, Imaging and Cognition Association of China” (VICA) was established.

**METHODS:**

VICA aims to recruit 10,000 participants, including 2000 with VRF, 3000 with CSVD, and 5000 stroke patients, to form a nationwide multicenter cohort. The study integrates clinical, neuroimaging, and multi‐omics data to better understand VCI heterogeneity, improve disease prediction, and ensure timely diagnosis.

**RESULTS:**

VICA has screened 2045 eligible VRF participants from six communities in Wuhan, Shanghai, and Taizhou, along with 602 CSVD and 1269 stroke patients from 135 hospitals nationwide. Baseline enrollment and follow‐up work are still ongoing.

**DISCUSSION:**

Establishing a high‐quality longitudinal cohort is crucial for understanding VCI pathogenesis and developing novel markers for early screening and diagnosis.

**Highlights:**

- Establish a large‐scale prospective longitudinal cohort comprising 10,000 participants, focusing on the high‐risk population of vascular cognitive impairment (VCI) in China.
- Establish a nationwide three‐tier medical network, make full use of resources, and achieve extensive enrollment of patients with cerebral small vessel disease and stroke patients.
- Utilize multimodal imaging and biomarkers to lay the foundation for constructing more‐precise risk models.
- Introduce eye movement and gait analysis as new methods for assessing cognitive function.
- Use positron emission tomography to further investigate the interaction between vascular factors and neurodegeneration.

## 1 BACKGROUND

### 1.1 VCI in China

Vascular cognitive impairment (VCI) encompasses a spectrum of cognitive decline attributable to vascular pathology to varying degrees of cognitive decline, ranging from subjective cognitive decline (SCD) and mild cognitive impairment (MCI) to dementia.^1^, ^2^, ^3^ Vascular dementia (VaD), the most severe form of VCI, is the second leading cause of dementia worldwide, accounting for 15%–30% of all cases.^4^, ^5^, ^6^ In China, the prevalence of VaD among individuals 60 years of age or older is ≈1.6%, affecting ≈3.92 million people.^7^, ^8^ According to the Global Burden of Disease Study 2019, China has the highest number of stroke patients globally, with 28.76 million cases, compared to 7.09 million in the United States and 12.23 million in Europe.^9^ Approximately one‐third of stroke patients develop post‐stroke cognitive impairment (PSCI), indicating that dementia related to stroke events is also the most prevalent in China.^10^ Consequently, research on VCI is essential to address the demands of national health policies aimed at alleviating this significant burden.[Fig alz14352-fig-0001]

### 1.2 The necessity of VCI research

Although VCI poses a significant threat to public health, its exact pathogenesis has not yet been fully elucidated and effective treatment for VCI is still missing.^11^, ^12^ VCI is widely recognized as a multifactorial disease involving genetic susceptibility,^13^ environmental factors, and a variety of vascular risk factors (VRF),^11^, ^14^, ^15^ leading to progressive deterioration of cerebrovascular conditions through a range of pathological changes.^15^, ^16^, ^17^, ^18^, ^19^, ^20^, ^21^ The deterioration begins with a vascular high‐risk state without obvious organic changes to a state of cerebral small vessel disease (CSVD), where the blood vessels exhibit chronic injury.^22^, ^23^ A decrease in brain reserve capacity can also trigger stroke events, leading to acute brain injury. In actual situations, cognitive impairment may occur at any of the aforementioned pathological stages, and different individuals may exhibit varying symptoms and disease trajectories.^24^ It is precisely because of the heterogeneity and complexity of VCI that the mechanisms of interaction between various factors and the relative contribution of each pathology have not yet been accurately defined. It is especially crucial to identify individuals exhibiting early cognitive impairment due to VRF, as these individuals face the greatest risk of progressing to VCI and stand to gain substantially from preventive measures.^11^, ^25^, ^26^

The employment of neuroimaging and biomarkers holds significant potential for future clinical research, enabling the early and often “silent” identification of individuals at risk for VCI. Imaging markers of CSVD, the location of cerebral infarcts, and the disconnection of brain networks provide possibilities for predicting VCI.^27^, ^28^, ^29^, ^30^ MarkVCID has identified 11 potential biomarkers related to small vessel disease (SVD)–associated cognitive impairment, presenting opportunities for further development and application.^11^, ^31^ It is imperative to solidify these identified biomarkers and to discover new ones to establish a reliable model for early identification of individuals at risk of VCI.^32^

**FIGURE 1 alz14352-fig-0001:**
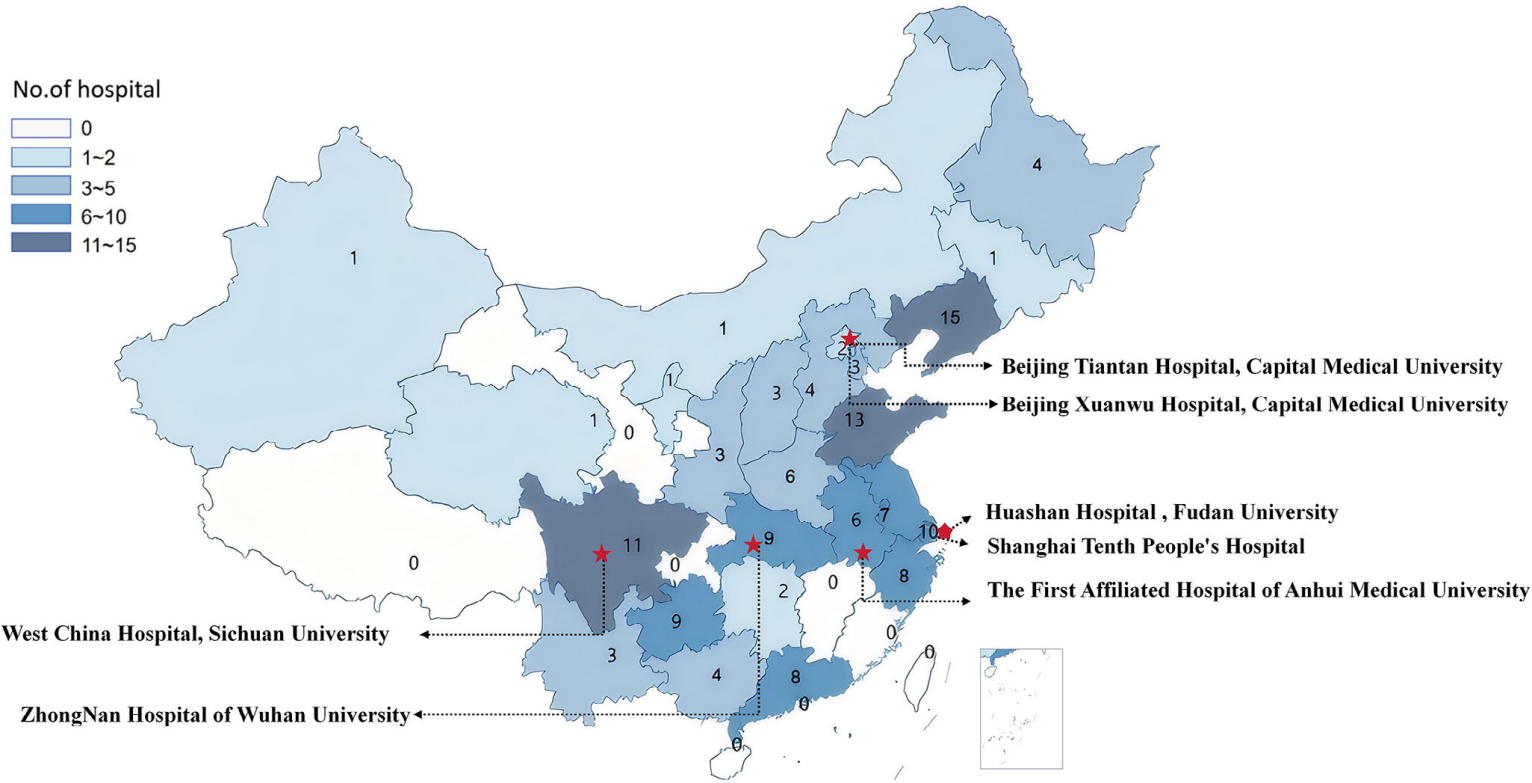
Map of the distribution of participating hospitals and seven quality control centers.

Therefore, establishing a large‐scale prospective cohort with uniform inclusion and exclusion criteria and standardized operating procedures is essential for studying VCI. Funded by the National Key Research and Development Program of China Ministry of Science and Technology, we have established an alliance in China named “Vascular, Imaging and Cognition Association of China” (VICA). VICA plans to enroll 10,000 high‐risk individuals for VCI from communities and 200 hospitals across the country. It focuses on establishing three distinct longitudinal cohorts: individuals with VRF, CSVD patients, and stroke patients. Up to now, 135 hospitals have joined this consortium (Figure 1).

**RESEARCH IN CONTEXT**

1. **Systematic review**: The authors searched PubMed for literature related to vascular cognitive impairment (VCI) or dementia. Due to the high heterogeneity of VCI, there are few whole‐course longitudinal studies based on various high‐risk populations for VCI in China.
2. **Interpretation**: It is necessary to establish a longitudinal cohort of Chinese VCI, to provide population resources for basic research, cohort studies, and clinical translation; to promote early diagnosis and treatment of VCI; and to establish a tiered diagnostic and treatment system. We have established an alliance (Vascular, Imaging and Cognition Association of China [VICA]) to jointly build a high‐quality VCI cohort and have described the research design and data collection of this comprehensive longitudinal database.
3. **Future directions**: VICA provides the foundation for exploring pathogenesis, identifying risk factors, establishing early warning models, and developing intervention and treatment measures. This is an initial effort, and the future goal is to expand the research impact by establishing cooperation with other research consortia and longitudinal studies and sharing data and resources, to promote progress in the global field of VCI prevention and treatment.

## 2 METHODS

### 2.1 Scientific objectives

VICA aims to establish a new framework for VCI by integrating clinical risk factors, neuroimaging, and omics data, to better understand its heterogeneity, and enable early screening, disease prediction, and timely diagnosis.[Fig alz14352-fig-0002]

1. Aim of the vascular risk factors (VRF) cohort: Construct a vascular cognitive impairment (VCI) community early‐screening procedure combining simple peripheral blood and digital biomarkers. Establish an early warning VCI model integrating omics data, imaging, and clinical information.
2. Aim of cerebral small vessel disease (CSVD) cohort: Explore microstructural and functional imaging techniques that outperform traditional magnetic resonance imaging (MRI) parameters, along with key biomarkers that more precisely capture small vessel pathological damage. Shed light on the interactions of the different molecular processes that help diagnosis, classification, and new therapeutic targets on VCI by integrating multi‐omics data sets.
3. Aim of Stroke cohort: Investigate the profound impact of stroke on cognitive function, including the effects of infarction location, size, brain network disconnection, and cerebral perfusion reduction on the progression of post‐stroke cognitive impairment (PSCI). Explore the relative contributions of neurodegenerative and vascular factors in the development and progression of PSCI.

### 2.2 Overview of VICA

**FIGURE 2 alz14352-fig-0002:**
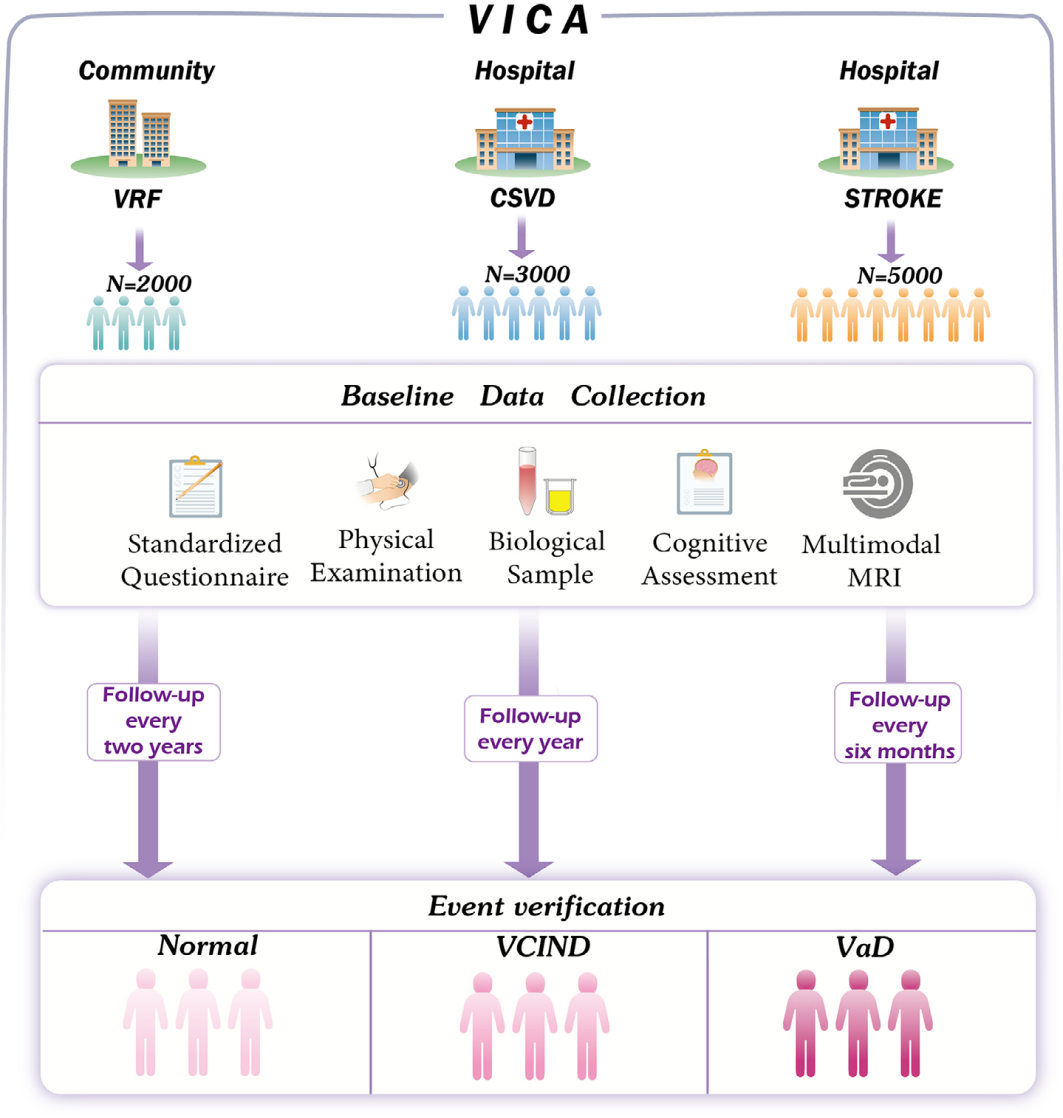
VICA study schema. CSVD, cerebral small vascular disease; MRI, magnetic resonance imaging; VaD, vascular dementia; VCIND, vascular cognitive impairment with no dementia; VICA, Vascular, Imaging and Cognition Association of China; VRF, vascular risk factor.

VICA is a prospective, multicenter, observational cohort study that leverages community and hospital resources to create a comprehensive research network. This study aims to recruit 2000 VRF individuals from the community and a total of 3000 CSVD patients and 5000 stroke patients from 200 hospitals. All participants will undergo clinical data collection, cognitive assessments, cranial magnetic resonance imaging (MRI) acquisition, and biological sample preservation (Figure 2). Specific research subgroups will be subjected to advanced MRI sequences or positron emission tomography (PET) imaging, multi‐omics analysis of biological samples, and multidimensional auxiliary assessments tailored to their research focus.

To accommodate the varying rates of cognitive decline among different participant groups, VICA has established a differentiated follow‐up schedule based on the distinct disease characteristics of the three cohorts. Follow‐up content includes physical examinations, cognitive assessments, biological sample collection, and imaging studies.

Each center participating in VICA will adhere to a unified set of standard operating procedures (SOPs) to ensure consistency and comparability in data collection, thereby safeguarding the scientific rigor and accuracy of the research findings.

### 2.3 Participants enrollment

We summarize the key inclusion criteria for the three cohorts, with detailed inclusion and exclusion criteria provided in Table S1. In addition, the power calculation used to determine the sample size is included.

#### 2.3.1 VRF participants

1. Age: 50–75 years of age;
2. Have at least one of the three major VRFs (hypertension, hyperglycemia, or dyslipidemia);
3. Without cancer, history of traumatic brain injury, significant organ failure, of serious psychiatric disorders, and have a life expectancy of more than 3 years.

#### 2.3.2 CSVD participants

1. Age: 50–80 years of age;
2. Meet the diagnostic criteria for CSVD: Moderate to severe white matter lesions (Fazekas score >1 in deep white matter or >2 in periventricular white matter), or mild white matter lesions (Fazekas score = 1 in deep white matter or = 2 in periventricular white matter) combined with lacunar infarcts or microbleeds;^33^
3. Have not had a stroke in the past 6 months.

#### 2.3.3 Stroke participants

1. Age: 18–80 years of age;
2. Ischemic stroke or transient ischemic attack (TIA);
3. Within 14 days from the onset of symptoms to enrollment;
4. Without a history of intravenous thrombolysis, intra‐arterial embolism, or bridging therapy within the time window of the acute phase.

### 2.4 Center selection

#### 2.4.1 Community sites for VRF cohort

The VRF cohort is drawn from the Jing'an Temple neighborhood and the Hongmei Road neighborhood in Shanghai, the Fruit Lake neighborhood in Wuhan, and three villages in Taizhou—Hutou, Caixiang, and Lubao—ensuring a mix of urban and rural demographics. Our preliminary work indicated that these areas have a dense population of middle‐aged and elderly individuals who are highly cooperative and capable of engaging in long‐term follow‐up studies.^34^, ^35^

#### 2.4.2 Hospital centers for CSVD and Stroke‐cohorts

Hospital centers are selected nationwide based on geographic distribution and population characteristics, with priority given to those centers with adequate research staff, relevant experience, and qualified equipment. These centers now span most regions of China, including multiple centers in the southeast coastal areas, the west, and the central parts of the country. This broad geographic coverage ensures a comprehensive and representative sample, reflecting the diversity of the national population and enhancing the generalizability of the VICA's findings.

The hospital centers are organized into a three‐tier medical network, classified according to their facilities, resources, and imaging capabilities. Seven hospitals, equipped with advanced medical technologies and expertise in data management, are designated for quality control. First‐tier centers focus on broad patient enrollment and monitoring for CSVD and stroke, meeting basic requirements for imaging and biosample collection. Second‐tier centers are tasked with collecting comprehensive clinical data, performing detailed cognitive assessments, conducting biomarkers assays, and utilizing advanced imaging techniques. Third‐tier centers integrate multi‐omics research, digital biomarkers studies, and advanced imaging methods such as functional MRI (fMRI) and PET scans, aiming to capture detailed participant information, achieve high follow‐up rates, and support the development of a high‐quality cohort for subgroup analysis.

### 2.5 Baseline data collection

#### 2.5.1 Ethical review and informed consent

This study has received ethical approval from Huashan Hospital, Fudan University, and subcenters initiating enrollment upon receiving their respective approvals. All participants provided written confirmation of consent, either personally or through a legally authorized representative.

#### 2.5.2 Questionnaires and neuropsychological assessment

Participants complete a standardized questionnaire administrated by trained personnel to obtain data on demographics, lifestyle, education, occupational status, medical and medication history, family history, and more (Table S2). The baseline physical examination, blood pressure, and clinical examination are conducted in person by experienced technicians at each central hospital.

Cognitive assessments are conducted by professionally trained evaluators using assessment batteries tailored specifically to each cohort (Table S3). These assessments encompass cognitive screening, global cognition, and comprehensive evaluations across multiple cognitive domains including language, executive functions/attention, memory, and visuospatial abilities. Mental and emotional states are also evaluated. VICA employs a variety of scale combinations designed to accommodate the unique characteristics of different participants, such as dominant hand paralysis, aphasia, and neglect, ensuring the assessments are broadly applicable.

#### 2.5.3 Biological samples

The biological samples of the participants will be collected for subsequent biochemical tests and subgroup genetic and biomarker studies. Certified nurses collect blood and urine samples according to standard procedures at baseline and during follow‐ups, with participants required to fast beforehand. Serum, plasma, and white blood cells are separated on site to minimize intermediate steps and reduce the risk of hemolysis. Biological specimens are stored in cryotube at −80°C, with regular cold‐chain shipments to nearby laboratories for analysis.

#### 2.5.4 Neuroimaging assessment

All participants will undergo a multimodal brain MRI scan on the same 3.0 T scanner at baseline and during each follow‐up visit, following a predetermined protocol. Participants undergo MRI scans including 3D‐T1, T2‐weighted imaging (T2WI), fluid‐attenuated inversion recovery (FLAIR), susceptibility‐weighted imaging (SWI), diffusion‐weighted imaging (DWI), and advanced sequences such as 3D‐pulsed continuous arterial spin labeling (3D pCASL), diffusion tensor imaging (DTI), magnetic resonance angiography (MRA), fMRI, and PET in some study sites. Specific parameters for each sequence are detailed in Tables S4 and S5.

### 2.6 Follow‐up arrangement and data collection

For the VRF and CSVD populations, the progression of cognitive impairment is relatively slow, thus requiring long‐term follow‐up observations. Therefore, we have planned follow‐ups for individuals with VRFs every 2 years and for CSVD patients annually. Each follow‐up visit includes neuropsychological assessments, physical examinations, biological sample collections, and imaging assessments.

For stroke patients, we have scheduled two follow‐up visits: at 6 months and 1 year post‐enrollment. Physical examinations and biological sample collections are conducted at both follow‐up visits. At the 6‐month visit, a full battery of cognitive assessments is performed to diagnose PSCI.^36^ At the 1‐year visit, in addition to the previously mentioned examinations, we also collect imaging data to observe changes in brain structure and vascular status.

At every follow‐up time point, the investigator of each center will contact their participants by telephone to arrange their re‐examination at the initial center. However, if individuals with VRF from the community develop brain imaging changes consistent with CSVD diagnostic criteria or experience a stroke, they may be transitioned to a hospital‐based cohort within the VICA network for further enrollment and follow‐up.

### 2.7 Data management

VICA has established an electronic data exchange system and a clinical research data management platform (CTDMP) utilizing a standardized public common data element (CDE) dictionary. This system facilitates online access to patient data, medications, imaging, and cognitive assessments, with cloud storage enabling remote quality control and information management, thereby reducing the risk of data loss and recording errors associated with paper records. VICA requires centers to record and audit all questionnaires and neuropsychological assessments on‐site. Centers must submit participant lists monthly, enter data into the system, and send digital imaging and communications in medicine (DICOM) image files for regular review by the quality control center.

### 2.8 Subgroups of the VICA study

Considering the large sample size and the diverse participating centers in the VICA study, it is impractical to require each site to collect complete biological samples and perform advanced multimodal brain imaging. Thus, specific subgroups have been formed to address distinct and innovative scientific hypotheses related to different forms of VCI.

#### 2.8.1 Subgroups aiming at early screening for VCI in VRF populations

- Genomics and metabonomics

Identifying specific biomarkers indicative of early vascular damage and cognitive changes is crucial for improving VCI screening and supporting the development of more accurate prevention strategies for high‐risk individuals. Peripheral blood was collected from community‐based VRF individuals. Genomic DNA is extracted and purified from whole blood and genotyped on an Axiom Precision Medicine Research Array (PMRA). Approximately 800,000 custom single nucleotide polymorphism (SNP) markers are selected for whole‐genome imputation. Nontargeted serum metabolomics analysis is conducted using a nuclear magnetic resonance (NMR) platform, with about 350 metabolic features and ratio parameters being quantified simultaneously.^37^

- Digital biomarkers

In community‐based cohorts, we have established a subgroup focused on eye movement and gait analysis, aiming to elucidate how digital biomarkers can enhance early cognitive screening for VRF individuals. Eye movement assessment, performed by trained professionals using an eye‐tracking system, includes tasks such as smooth pursuit, anti‐saccade, and visual paired comparison.^38^ Gait assessment involves the Timed Up and Go (TUG) test, the Tinetti test, and other quantitative methods utilizing wearable sensors and motion capture systems.^39^ These noninvasive markers, measured through wearable devices, are safe, easy to implement, and culturally adaptable, making them ideal for community‐based screening.

#### 2.8.2 Subgroups aiming at accurate diagnosis and classification in patients with CSVD

- Early microstructural changes in CSVD populations

The heterogeneous nature of cerebrovascular disease makes it challenging to fully elucidate the pathological substrates of VCI using traditional imaging markers. Thus subgroups with more advanced MRI sequences were established. Through the observation of microvascular changes, blood–brain barrier damage, and cerebral blood flow alterations, we aim to deepen our understanding of VCI pathogenesis and to discover new imaging biomarkers, thereby improving the accuracy of VCI diagnosis and classification. The imaging sequences performed by these centers include 3D‐T1, FLAIR, T2WI, SWI, MRA, 3D pCASL, DTI, and fMRI. Participants in the subgroup are required to undergo scans with these sequences at baseline and during each follow‐up.

- Integrated multi‐omics analysis

CSVD is highly heterogeneous, encompassing various pathological mechanisms such as hypertension‐related arteriolosclerosis, cerebral amyloid angiopathy, and venous abnormalities. To identify complex molecular signatures associated with CSVD, it is significant to take an integrated systems analysis that combines multi‐omics data to highlight the interrelationships among the involved pathways.

Multi‐omics data, such as proteome, lipid metabolomics, and exosome transcriptome were collected and analyzed in a subset of CSVD patients. The iCluster method is planned to be used for integrative clustering from multiple data types. By integrated multi‐omics analysis, we aim to further elucidate the mechanisms underlying VCI and select promising biomarkers as an integrated biomarker panel to enhance diagnostic accuracy.

#### 2.8.3 Subgroups aiming at predicting the occurrence and prognosis of PSCI in stroke patients

- Peripheral blood biomarkers

Baseline and follow‐up blood samples were collected from stroke patients at second‐ and third‐tier centers. Biomarkers, including but not limited to amyloid beta (Aβ), phosphorylated tau‐217 (p‐tau217), neurofilament light (NfL), glial fibrillary acidic protein (GFAP), monocyte chemoattractant protein‐1 (MCP‐1), matrix metalloproteinases (MMPs), interleukin‐6 (IL‐6), brain‐derived neurotrophic factor (BDNF), vascular endothelial growth factor (VEGF), and placental growth factor (PLGF), will be determined using mass spectrometry, enzyme‐linked immunosorbent assay (ELISA), and single‐molecule array (Simoa).

By analyzing the correlation of these markers and cognitive outcomes, we aim to further elucidate the mechanisms underlying PSCI and select biomarkers to predict the occurrence and prognosis of PSCI in stroke patients.

- Brain imaging markers

The impact of stroke on cognitive impairment will be investigated in detail using multimodal brain MRI. The sequences included were listed as follows: structural MRI for brain structural analysis, DWI for infarction signature analysis, DTI for microstructural analysis, and 3D pCASL for perfusion analysis. These data will be analyzed using established automated pipelines. These neuroimaging tools will play an important role in predicting poststroke cognitive recovery.

- The contribution of neurodegeneration in VCI

In third‐tier centers, stroke patients with good compliance are selected to undergo PET scans using fluorodeoxyglucose (FDG), amyloid, and/or tau as the main tracers. The results of the PET scans are subjected to calculations of standardized uptake values, image fusion, and quantitative analysis assessments, to explore whether neurodegeneration accelerates the progression of PSCI and to assess the independent and combined effects of existing CSVD, stroke, and Alzheimer's disease (AD) pathology on VCI.

## 3 RESULTS

### 3.1 Center construction status

We have selected six communities in Shanghai, Taizhou, and Wuhan for screening and enrolling the VRF population. These communities have previously established large community‐based cohorts and have stable resident populations with a low rate of follow‐up loss, ensuring a sufficient number of VRF participants for the VICA study.

For the CSVD and Stroke cohorts, our recruitment plan includes 120 first‐tier centers, 60 second‐tier centers, and 20 third‐tier centers. To date, we have completed the recruitment at 135 centers. Of these, 50 centers are enrolling outpatients with CSVD, consisting of 30 first‐tier, 15 second‐tier, and 5 third‐tier. In addition, 90 centers are enrolling hospitalized stroke patients, including 57 first‐tier, 28 second‐tier, and 5 third‐tier centers. Five third‐tier centers are simultaneously building cohorts for CSVD and STROKE, whereas other centers are only building one of the cohorts. Approximately 400 CSVD patients and 900 stroke patients can be enrolled each month. Hospital centers are continuing active recruitment efforts to expand geographic coverage and population diversity, aiming to complete baseline enrollment as soon as possible. Details regarding the community sites and the construction of hospital centers are provided in the Supplementary Materials.

### 3.2 Characteristics of VICA participants

Currently, 2045 individuals meeting the inclusion criteria of the VRF cohort have been selected from previously established community‐based cohorts (Table 1). Overall, 55.7% of the participants are female, and the median age of all individuals is 64 years. Participants with two or more VRFs accounted for 42.6%. The Mini‐Mental State Examination (MMSE) median score was 28.

**TABLE 1:** Baseline characteristics of the participants in the VICA‐VRF.

(*n* = 2045)	
Age (years)	64 (61, 69)
Female	1140 (55.7)
BMI, kg/m^2^ ^a^	24.6 (22.7–26.9)
Education level^a^	
Illiteracy	221 (10.3)
Elementary school	329 (16.0)
Middle school	419 (20.4)
High school	495 (24.2)
University or above	585 (28.6)
Blood pressure, mmHg^a^	
Systolic	143 (130, 157)
Diastolic	80 (73, 88)
Number of VRFs	
1	1171 (57.2)
2	728 (35.5)
3	146 (7.1)
MMSE score^a^	28 (27, 29)

*Note*: Data represent *n* (%) or median (interquartile range).

Abbreviations: BMI, body mass index; MMSE, Mini‐Mental Status Examination; VICA, Vascular, Imaging and Cognition Association of China; VRFs, vascular risk factors.

^a^ The missing values of BMI, education level, blood pressure, and MMSE score are 4, 6, 4, and 1, respectively.

To date, 602 CSVD patients have been enrolled and passed quality control (Table 2). The study population consists of a diverse group, with 36.48% being female, and a median age of 63 years. Among the participants, 21.5% were current smokers, and 18.4% consumed alcohol. Moreover, 66.1% had been diagnosed with hypertension. Lacunar infarcts were present in 51.9% of participants, whereas cerebral microbleeds were observed in 24.0%. The median MMSE and Montreal Cognitive Assessment (MoCA) scores were 26 and 21, respectively.

**TABLE 2:** Baseline characteristics of the participants in the VICA‐CSVD.

(*n* = 602)	
Characteristics	
Age, years	63 (59, 68)
Female	288 (47.8)
BMI, kg/m^2^ ^b^	24.1 (22.2, 26.1)
Current smokers^b^	130 (21.5)
Current drinkers^b^	111 (18.4)
Blood pressure, mmHg^b^	
Systolic	137 (125, 150)
Diastolic	81 (74, 90)
Education level^b^	
Illiteracy	98 (16.3)
Elementary school	127 (21.1)
Middle school	175 (29.1)
High school	116 (19.3)
University or above	74 (12.3)
Medical history	
Hypertension	398 (66.1)
Diabetes mellitus	219 (36.3)
Hyperlipidemia	96 (15.9)
CSVD markers	
Lacuna	313 (51.9)
Cerebral microbleeds^b^	270 (44.8)
WMHs^a^	
Periventricular^b^	2 (1, 2)
Deep^b^	2 (1, 2)
MoCA score^b^	21 (15, 25)
MMSE score^b^	27 (23, 29)

*Note*: Data represent *n* (%) or median (interquartile range).

Abbreviations: BMI, body mass index; CSVD, cerebral small vascular disease; MMSE, Mini‐Mental Status Examination; MoCA, Montreal Cognitive Assessment; VICA, Vascular, Imaging and Cognition Association of China; WMH, white matter hyperintensity.

^a^ WMH was estimated using Fazekas score.

^b^ The missing values of BMI, current smokers, current drinkers, blood pressure, education level, cerebral microbleeds, periventricular WMHs, deep WMHs, MoCA score, and MMSE score are 17, 11, 12, 15, 12, 1, 34, 68, 43, and 12, respectively.

Currently, VICA has successfully recruited 1269 ischemic stroke or TIA patients and collected their baseline data (Table 3). Baseline enrollment for stroke is expected to continue for another 9 months. Of the enrolled patients, 27.1% were women with a median age of 63 years. Among them, 33.6% were current smokers and 24.2% consumed alcohol, 14.8% had a history of stroke and 71.7% had developed hypertension. The median MMSE and MoCA scores were 26 and 23, respectively. Furthermore, 278 ischemic stroke or TIA participants have completed their first follow‐up assessment, with a PSCI incidence rate of 33.45% and a dementia incidence rate of 15.83%.

**TABLE 3:** Baseline characteristics of the participants in the VICA‐Stroke.

(*n* = 1269)	
Characteristics	
Age, years	63 (55, 70)
Female	345 (27.1)
BMI, kg/m^2^ ^a^	25.1 (22.7, 27.4)
Current smokers^a^	427 (33.6)
Current drinkers^a^	308 (24.2)
Education level^a^	
Illiteracy	69 (5.4)
Elementary school	224 (17.7)
Middle school	422 (33.3)
High school	287 (22.6)
University or above	191 (15.1)
Medical history	
Previous stroke^a^	189 (14.8)
Hypertension	911 (71.7)
Diabetes mellitus	463 (36.4)
Hyperlipidemia	307 (24.1)
Atrial fibrillation^a^	134 (10.5)
TOSAT classification^a^	
Large artery atherosclerosis	583 (45.9)
Cardioembolism	37 (2.9)
Small vessel occlusion	470 (37.0)
Other determined etiology	29 (2.3)
Undetermined etiology	93 (7.3)
OCSP classification^a^	
Total anterior circulation infarcts	72 (5.7)
Partial anterior circulation infarcts	630 (49.6)
Lacunar circulation infarcts	185 (14.6)
Posterior circulation infarcts	299 (23.6)
NIHSS	2 (1, 4)
MoCA score^a^	23 (17, 26)
MMSE score^a^	26 (23, 28)

*Note*: Data represents a number (%) or median (interquartile range).

Abbreviations: BMI, body mass index; MMSE, Mini‐Mental Status Examination; MoCA, Montreal Cognitive Assessment; NIHSS, National Institute of Health Stroke Scale; OCSP, Oxfordshire Community Stroke Project; TOAST, Trial of Org 10172 in Acute Stroke Treatment; VICA, Vascular, Imaging and Cognition Association of China.

^a^ The missing values of BMI, current smokers, current drinkers, education level, previous stroke, atrial fibrillation, TOSAT classification, OCSP classification, MoCA score, and MMSE score, are 75, 33, 33, 76, 2, 2, 57, 83, 317, and 194.

### 3.3 Subgroup research progress

Within the VRF cohort, we have collected biological samples including blood and urine from 2000 individuals, and have completed metabolomics analyses on 1605 samples and genomics analyses on 603 samples. In addition, ≈1400 individuals have finished the assessment of digital biomarkers.

Baseline serum biomarker testing has been performed on approximately ≈800 stroke patients. Advanced MRI scans of 1000 stroke and 300 CSVD patients have been analyzed for microvascular changes, blood–brain barrier damage, and cerebral blood flow alterations. In addition, the PET subgroup in Shanghai has been successfully initiated, with 100 stroke patients and 80 CSVD patients having completed baseline PET scans. The detailed measurements and enrollment progress of subgroup analyses are presented in Table S6.

## 4 DISCUSSION

### 4.1 The innovation of VICA

The VICA study is designed to include three key population groups representing distinct stages of VCI: VRF, CSVD, and stroke. The VRF cohort is sourced from well‐established, community‐based cohorts with low attrition rates, encompassing both urban and rural populations to reflect China's demographic diversity. Participants for the CSVD and stroke cohorts are recruited from hospitals within a nationwide, three‐tier medical network. Given the large baseline sample size, advanced imaging and omics testing cannot be performed on all participants. To address this limitation, we have developed subgroup studies that incorporate detailed, multidimensional data collection, including advanced structural and functional imaging, as well as molecular imaging for select populations. This approach aims to enhance the precision of VCI disease modeling and framework refinement.

For individuals with VRF, employing simple and noninvasive methods for the early prediction and identification of VCI is essential. Although cranial MRI provides extensive insights into brain structural changes, its implementation in community settings poses significant challenges. Detailed cognitive assessments, although effective, are time‐consuming and labor‐intensive. Factors such as China's large population, regional dialects, and lower educational attainment further complicate their widespread use. In contrast, blood biomarkers present a high degree of accessibility, cost‐effectiveness, and stability, making them ideal for early VCI screening in community populations with VRF.^40^

Furthermore, advancements in artificial intelligence (AI) and virtual reality (VR) have facilitated the emergence of digital biomarkers—such as gait analysis and eye movement tracking—as promising tools for early dementia diagnosis.^41^, ^42^ These noninvasive markers, measured through wearable devices, are safe, easy to implement, and culturally adaptable, rendering them suitable for community screening. A study conducted in China identified significant differences in 14 eye movement parameters and 32 gait features between individuals with normal cognition and those with cognitive impairment.^43^ The dual‐task methodology combining eye movement and gait analysis demonstrated superior efficacy in distinguishing normal cognition from cognitive impairment compared to traditional assessments such as the MMSE and MoCA. Consequently, VICA has designed eye and gait tests to investigate these emerging cognitive detection tools and evaluate their potential in predicting long‐term cognitive progression in VRF patients. Through the analysis of multidimensional markers, we aim to identify early vascular damage and cognitive changes, thereby enhancing VCI screening and supporting the development of targeted prevention strategies for high‐risk individuals.

In CSVD, the severity of cranial imaging findings often does not fully correspond to clinical symptoms, making it challenging to accurately predict both imaging and cognitive progression in clinical practice. Currently, the diagnosis of CSVD relies primarily on cranial MRI, which does not directly visualize small vessels but rather captures brain tissue damage attributed to CSVD, such as infarcts, white matter hyperintensities, and microbleeds.^36^ These conventional imaging markers show significant variability among patients, limiting their effectiveness in predicting the onset and progression of VCI.^44^ Advanced imaging techniques, such as DTI, provide greater sensitivity than fluid‐attenuated inversion recovery (FLAIR) by assessing white matter structural integrity. Perfusion MRI allows for the quantification and visualization of blood–brain barrier disruption and cerebral blood flow, whereas functional MRI offers more detailed insights into brain microstructure and function.^44^

Beyond the need for more advanced imaging, CSVD research must address key pathological changes, including vascular wall damage, blood–brain barrier disruption, and imbalances between perfusion and metabolic demand, which often remain undetected.^45^, ^46^ This gap frequently results in notable discrepancies between clinical symptoms and imaging findings in previous CSVD studies. Identifying early, potentially reversible stages of CSVD holds significant clinical value. Thus, there is a critical need for new classification and prognostic models that integrate multi‐omics biomarkers reflecting vascular pathophysiology with more sensitive imaging markers of microstructural alterations.

In constructing the stroke cohort, previous studies frequently excluded patients with significant neurological deficits—such as dominant‐hand paralysis, aphasia, or hemianopia—who were unable to complete standard cognitive assessments. Consequently, these cohorts often consisted of milder stroke cases, failing to capture the full spectrum of cognitive impairment in the post‐stroke population. In our study, we employed neuropsychological scales tailored to the specific needs of different patient groups, ensuring broader and more representative inclusion of stroke patients.

It is also important to recognize the potential overlap and synergistic effects between cerebrovascular pathology and AD pathology, which may jointly drive cognitive decline.^47^, ^48^, ^49^ Vascular pathology and disruption of the blood–brain barrier can reduce Aβ clearance, leading to increased neurotoxicity.^50^, ^51^ The respective contributions of neurodegeneration, pre‐existing small vessel disease, and ischemic stroke severity to the onset and progression of PSCI remain insufficiently understood. Therefore, integrating peripheral blood biomarkers that reflect neurodegeneration, neurovascular injury, and neuroprotective factors, alongside PET imaging studies, is crucial for building a more comprehensive understanding of PSCI and developing robust cohorts for future research.

### 4.2 The future of VICA

VICA represents a large‐scale, multicenter prospective cohort study aimed at establishing a new framework for VCI. It conducts multi‐omics data exploitation based on bioinformatics modeling to continuously obtain reliable candidate molecular markers within the cohort and inform early clinical diagnosis and intervention strategies to mitigate cognitive decline. It can support randomized controlled trials (RCTs) for CSVD and PSCI to assess the impact of different pharmacological interventions on the cognitive function of CSVD and PSCI patients, as well as their potential benefits on the overall disease progression, providing new research methods for the treatment and prognosis of VCI.

Understanding the unique biological and environmental context of China, VICA will also explore the existence of biomarkers or treatment strategies specific to the Chinese population. This includes comparative studies between Chinese and Western databases or other ethnic groups, which will provide insights into potential cultural or genetic differences in VCI pathology.

In addition, by investigating the correlation between vascular factors and neurodegeneration, VICA is poised to uncover early markers and pathological mechanisms shared between VCI and AD, thereby aiding in the early screening and diagnosis of the condition. Scientists may also gain insights into how to reduce the incidence and delay the progression of AD by improving vascular health, offering potential targets for the development of novel interventional approaches.

Although our current sample size may not be optimal, we envisage progressively meeting the cohort's numerical prerequisites in the near future and will persist in advancing follow‐up assessments. Collaborative efforts among various centers nationwide ensure seamless participant enrollment and follow‐up procedures. We also look forward to collaborating with other alliances to expand our impact and provide necessary research resources to other researchers. We stand prepared to collaborate with domestic research teams to collectively address the substantial disease burden faced by China's aging populace. In addition, we remain receptive to international collaborations aimed at bridging research gaps within the field of VCI.

## CONFLICT OF INTEREST STATEMENT

The authors declare no conflict of interest. Author disclosures are available in the supporting information.

## CONSENT STATEMENT

The VICA study was reviewed and approved by ethics and safety review committees. All participants (or their legally authorized representatives) reviewed and signed an approved informed consent document.

## Supporting information



Supporting Information

Supporting Information
