# Supplementary material for: Imaging, biomarkers, and vascular cognitive impairment in China: Rationale and design for the VICA study
Source: Alzheimers Dement. 2024 Nov 13;20(12):8898–909. doi: 10.1002/alz.14352 (PMC11667496; doi:10.1002/alz.14352)
Supplement: Supplementary file 1 — Supporting Information [file ALZ-20-8898-s002.pdf]

Supplementary Materials

- Table 1-6
- Sample Size Calculation
- The complete list of VICA centers
- Ethic and Informed Consent
- Control Attrition
- Status of VICA Center Construction

Table 1 Inclusion and exclusion criteria of VRF, CSVD and stroke population

	Inclusion Criteria	Exclusion Criteria
**VRF**	-Age: 50-75 years old<br>-Have at least one of the three major vascular risk factors (Hypertension, Hyperglycemia, Dyslipidemia)*<br>-Be able to complete medical examinations such as questionnaire survey, physical examination, and head MRI<br>-Be willing to voluntarily participate in the study and follow-up assessments, provide biological samples, and sign an informed consent form	-Pregnancy, malignant tumors, failure of important organs or life expectancy of less than 3 years<br>-Diagnosed with dementia before stroke<br>-Congenital intellectual disability, history of traumatic brain injury or serious psychiatric disorders*<br>-Have a history of psychoactive substance abuse<br>-Severe visual or hearing impairment that prevent cooperation with neuropsychological assessment<br>-Other serious medical conditions that affect cognitive testing, such as coma, epilepsy, hypothyroidism symptoms, hypoxemia, etc.<br>-Contraindications to MRI examination<br>-Have imaging findings related to other central nervous system diseases* on MRI

	Inclusion Criteria	Exclusion Criteria
**CSVD**	-Age: 50-80 years old<br>-Meet diagnostic criteria of CSVD: Moderate to severe white matter lesions (Fazekas score >1 in deep white matter or >2 in periventricular white matter), or mild white matter lesions (Fazekas score = 1 in deep white matter or = 2 in periventricular white matter) combined with lacunar infarcts or microbleeds<br>-Be able to complete medical examinations such as questionnaire survey, physical examination, and head MRI<br>-Be willing to voluntarily participate in the study and follow-up assessments, provide biological samples, and sign an informed consent form	-Have a stroke event within the past six months<br>-Pregnancy, malignant tumors, failure of important organs or life expectancy of less than 3 years<br>-Congenital intellectual disability, history of traumatic brain injury or serious psychiatric disorders<br>-Have a history of psychoactive substance abuse<br>-Severe visual or hearing impairment that prevent cooperation with neuropsychological assessment<br>-Other serious medical conditions that affect cognitive testing, such as coma, epilepsy, hypothyroidism symptoms, hypoxemia, etc.

	Inclusion Criteria	Exclusion Criteria
**STROKE**	-Age: 18-80 years old<br>-Onset time: ≤14 days<br>-According to the 2013 U.S. New Definition of AHA/ASA Stroke, clinically diagnosed acute ischemic stroke cases: episodes of neurological dysfunction caused by focal brain, spinal cord or retinal infarction<br>-Be able to complete medical examinations such as questionnaire survey, physical examination, and head MRI<br>-Be willing to voluntarily participate in the study and follow -up assessments, provide biological samples, and sign an informed consent form	-Received intravenous thrombolysis, intraarterial embolism and bridging therapy within the time window of the acute phase<br>-Pregnancy, malignant tumors, failure of important organs or life expectancy of less than 3 years<br>-Diagnosed with dementia before stroke<br>-Congenital intellectual disability, history of traumatic brain injury or other serious psychiatric disorders<br>-Have a history of psychoactive substance abuse<br>-Severe visual or hearing impairment that prevent cooperation with neuropsychological assessment<br>-Other serious medical conditions that affect cognitive testing, such as coma, epilepsy, hypothyroidism symptoms, hypoxemia, etc.

*Definition of vascular risk factors:

Hypertension: Systolic blood pressure ≥140 mmHg or diastolic blood pressure ≥90 mmHg on two or more occasions; or diagnosed with hypertension; or currently taking antihypertensive medication.

Hyperglycemia: Fasting blood glucose ≥7.0 mmol/L; or diagnosed with diabetes; or currently using antidiabetic medication.

Dyslipidemia: Total cholesterol ≥5.2 mmol/L; or triglycerides ≥1.7 mmol/L; or diagnosed with hyperlipidemia; or currently taking lipid-lowering medication.

*Serious psychiatric disorders: Anxiety, depression, schizophrenia, bipolar disorder, intellectual disability, or any other diagnosed mental illnesses that require medication for control.

*Other central nervous system diseases: Parkinson's disease, epilepsy, myasthenia gravis, multiple sclerosis, motor neuron disease, immune-related brain and spinal cord disorders.

Table 2 Summary of measurements at baseline and follow-up in the VICA

Exposure Category	Variables or Measurements
**Questionnaire and Interview**	
**Demographics**	Sex; age; date of birth; nationality; marital status
**Socio-economic status**	Education; employment status (job type and physical labor); Individual and household incomes; health insurance coverage; housing condition (house area and house area per person); householder's education level; ownership of several household appliances (private car, motorbike, mono-television, color-television, washing machine, vacuum, refrigerator, freezer, computer, and bathroom)
**Personal health behavior**	Tobacco smoking; alcohol drinking; physical activity
**Medical history**	Medical conditions diagnosed by doctors; medication history; surgical history
**Family history**	Family history of hypertension; coronary artery disease; diabetes; stroke; dementia; atrial fibrillation; cancer and other chronic diseases
**Physical Measures**	
**Anthropometrics**	Standing height; weight; waist and hip circumference; height; weight; body mass index

**Blood pressure**	Two automated measures, 3-5 minute apart
**Neuroimaging**	Multimodal 3.0 T brain magnetic resonance imaging (MRI: GE; Siemens)
**Electrocardiography**	12-Lead electrocardiography (ECG-1350P, Nihon Kohden, Japan)
**Carotid artery ultrasound**	Carotid intima-media thickness; plaque; other carotid-related vascular phenotypes (Acuson S2000; Siemens AG, Munich, Germany)
**Clinical lab-based tests**	Morning fasting serum alanine transaminase; aspartate aminotransferase; direct bilirubin; total bilirubin; creatinine; urea nitrogen; uric acid; glucose; total cholesterol; triglycerides; high-density lipoprotein cholesterol; low-density lipoprotein cholesterol; fasting blood sugar; HbAlc; Homocysteine
**Biospecimen Collection**	Morning fasting blood (~15 mL); urine (~15 mL)
**Omics Data**	
**Genetics**	Genotyping on blood-derived genomic DNA by Axiom Precision Medicine Research Array (~ 800,000 custom single nucleotide polymorphisms)

**Metabolomics**	Serum metabolomic profiling performed by nuclear magnetic resonance (NMR) platform
**Proteomics**	Mass spectrometry, enzyme-linked immunosorbent assay (ELISA), and single-molecule array (Simoa)
**Auxiliary Assessment**	Gait; eye movement

Table 3 Selection of Neuropsychological Assessment Scales in VICA

<table>
<tr><th colspan="2" rowspan="2">Neuropsychological Assessment</th><th rowspan="2">VRF/CSVD</th><th colspan="3">STROKE</th></tr>
<tr><th>without aphasia, neglect or dominant hand paralysis</th><th>with dominant hand paralysis</th><th>with aphasia or neglect</th></tr>
<tr><td colspan="2">Screening</td><td>AD-8，NINDS-CSN 5min</td><td>AD-8， NINDS-CSN 5min ， IQCODE</td><td>AD-8, IQCODE</td><td>AD-8, IQCODE</td></tr>
<tr><td colspan="2">Global cognition</td><td>MMSE, MoCA</td><td>MMSE, MoCA</td><td>MMSE</td><td rowspan="5">OCS-P</td></tr>
<tr><td rowspan="4">Cognitive domain</td><td>memory</td><td>CAVLT</td><td colspan="2">CAVLT</td></tr>
<tr><td>Executive function/ processing speed</td><td>SDMT, CTT</td><td>SDMT, CTT</td><td>SCWT</td></tr>
<tr><td>visuospatial</td><td>CFT</td><td colspan="2">JLO</td></tr>
<tr><td>language</td><td>AFT, BNT-15</td><td colspan="2">AFT, BNT-15</td></tr>
<tr><td colspan="2">Function</td><td colspan="4">FAQ</td></tr>
<tr><td colspan="2">Mental Health</td><td colspan="4">HAMA, HAMD，PHQ-9, GAD-7, NPI-Q</td></tr>
</table>

**The individuals in STROKE cohort do not undergo the cognitive domain assessment at baseline.*

Abbreviations: MMSE, Minimum Mental State Examination; MoCA, Montreal Cognitive Assessment; OCS-P, The Chinese (Putonghua) version Oxford Cognitive Screen; CAVLT, Chinese auditory verbal learning test; SDMT, Symbol Digit Modalities Test; SCWT, Stroop Color-Word Test; CTT, Color Trails Test; JLO, Judgement of Line Orientation; AFT, Animal Fluency Test; BNT-15, Boston Naming Test; CFT, Rey-Osterrieth Complex Figure Test; HAMA, Hamilton Anxiety Scale; HAMD, Hamilton Depression Scale; PHQ-9, Patient Health Questionnaire-9; GAD-7, Generalized Anxiety Disorder 7-item; NPI-Q, Neuropsychiatric inventory questionnaire; FAQ, Functional Activities Questionnaire; IQCODE, Informant Questionnaire on Cognitive Decline in the Elderly.

Table 4 Neuroimaging scan sequences in VICA

**Community Cohort**	**Hospital Cohort**		
**VRF**		**CSVD**	**STROKE**
T1WI, T2WI, FLAIR, SWI	**First-Tier**	T1WI, FLAIR, T2WI, SWI	T1WI,T2WI,FLAIR,DWI,SWI
	**Second-Tier**	3D-T1, FLAIR, T2WI, SWI, 3D pCASL, DTI, MRA	3D-T1, FLAIR, DWI, T2WI, SWI, 3D pCASL, DTI, MRA
	**Third-Tier**	3D-T1, FLAIR, T2WI, SWI, 3D pCASL, DTI, MRA, fMRI, PET	3D-T1, FLAIR, DWI, T2WI, SWI, 3D pCASL, DTI, MRA, fMRI, PET

Abbreviations: T1WI, T1-weighted imaging; T2WI, T2-weighted imaging; FLAIR, fluid-attenuated inversion recovery; SWI, susceptibility weighted imaging; DWI, diffusion weighted imaging; 3D pCASL, 3D pseudo-continuous arterial spin labeling; DTI, diffusion tensor imaging; fMRI, functional magnetic resonance imaging; 3D-T1, 3D T1-weighted imaging; MRA, magnetic resonance angiography; PET, positron emission tomography.

Table 5 Parameters of MRI scan sequences in VICA

MRI sequence	TR (ms)	TE (ms)	Flip Angle (°)	Slice Thickness
**3D T1**	7.7	3.1	12	1.0
**T1WI**	1450.0	25.2	111	4.0
**T2WI**	4271.5	91.0	142	6.0
**FLAIR**	8000.0	113.5	160	2.0
**SWI**	54.0	23.5	15	2.0
**fMRI**	2000.0	30.0	70	4.0
**DTI**	13000.0	96.4	90	2.5
**DWI**	4500.0	97.5	90	4.0
**3D pCASL(PLD=1.5 s)**	4611.0	10.5	111	4.0
**3D pCASL(PLD=2.5 s)**	5306.0	10.5	111	4.0
**MRA**	21.0	3.6	18	0.6

Abbreviations: T1WI, T1-weighted imaging; T2WI, T2-weighted imaging; FLAIR, fluid attenuated inversion recovery; SWI, susceptibility weighted imaging; BOLD-fMRI, blood oxygenation level dependent functional magnetic resonance imaging; DTI, diffusion tensor imaging; DWI, diffusion-weighted imaging; 3D pCASL, 3D pulsed continuous arterial spin labeling; PLD, post labeling delay; MRA, magnetic resonance angiography; TE, echo time; TR, repetition time.

Table 6 Measurements and enrollment progress of subgroups of VICA

	Items	Number
**Advanced Neuroimaging**	CSVD:<br>Structural and functional brain phenotypes, including gray matter structure, white matter microstructure, cerebral blood flow, and brain FC.<br>STROKE:<br>Structural and functional brain phenotypes, including gray matter structure, white matter microstructure, cerebral blood flow, brain FC, and stroke lesions.	CSVD: N = 300<br>STROKE: N = 1,000
**PET**	Standardized uptake value; tracer network characteristics	CSVD: N = 80<br>STROKE: N = 100
**Metabolomics**	210 types of absolutely quantified metabolite indicators; 144 types of ratio or percentage indicators between metabolites	VRF: N = 1,605
**Genomics**	GWAS framework loci: 568,000<br>Clinical research exon loci: 138,000<br>Cohort loci in the population: 21,000<br>Special genetic regions such as chrY, Mt, and HLA: 50,000	VRF: N = 603
**Proteomics**	P-tau217, Aβ, GFAP, NFL, BDNF, VEGF, PLGF, MCP-1, IL-6, etc.	STROKE: N = 600
**Digital biomarkers**	105 features of gait and eye movement, including speed, stride length, smooth pursuit, regressive saccades, etc.	VRF: N = 1,400

Abbreviations: BDNF, brain-derived neurotrophic factor; chrY, chromosome Y; CSVD, cerebral small vascular disease; FC, functional connectivity; GFAP, glial fibrillary acidic protein; GWAS, genome-wide association studies; HLA, Human Leukocyte Antigen; IL-6, Interleukin-6; MCP-1, monocyte chemoattractant protein-1; Mt, mitochondrial DNA; NFL, neurofilament light; PET, positron emission tomography; PLGF, placental growth factor; VEGF, vascular endothelial growth factor; VRF, vascular risk factor

Sample Size Calculation

For the VRF population, the hazard ratio (HR) for digital biomarkers—gait speed is 2.28 (J Gerontol A Biol Sci Med Sci. 2017 May 1; 72(5): 655-661), for the biomarker IGF-1 is 2.19 (Psychoneuroendocrinology. 2017 Dec: 86: 169-175), for WMH volume and dementia onset is 1.68 (Alzheimers Dement. 2023 Dec;19(12):5632-5641); in a 10-year follow-up of community-dwelling elderly, the incidence of dementia is 22.9% (Alzheimers Dement. 2023 Dec; 19(12): 5632-5641). With a two-sided test level α set at 0.05 and a power of the test (1-β) at 0.90, the PASS software was used to calculate the required sample sizes at baseline to be 546, 624, and 1382 individuals, respectively. Considering subsequent attrition and omics data quality control, an additional 20% of the sample size is added to the maximum required, totaling 1659 individuals. The current sample size of 2000 individuals is sufficient for the study needs.

For the CSVD population, the risk ratio (OR) for the biomarker IL-6 in relation to small vessel disease-related cognitive impairment is 2.04(European Journal of Neurology 2016, 23: 656–663); the OR for the CSVD burden score and cognitive impairment is 1.593 (Front Neurol. 2022 Aug 11:13:944205), as per the 14-year follow-up study on Cerebral Small Vessel Disease Progression and the Risk of Dementia by RUN DMC, where 20% of CSVD patients developed dementia. Using the PASS software (Version 2021), with a two-sided test level α set at 0.05 and a test power (1-β) set at 0.90, the aforementioned parameters were substituted into the Cox proportional hazards regression model. The required sample size at baseline was calculated to be 434 and 1995 individuals. Considering subsequent attrition and omics data quality control, an additional 20% of the sample size is added to the maximum required, totaling 2394 individuals. The current sample size of 3000 individuals is sufficient for the study needs.

For stroke population, the OR for the biomarker ptau181 of PSCI is 0.63 (Front Aging Neurosci. 2022 Apr 29:14:889101.), and the OR for the imaging predictive indicator—the network impact score is 1.27 (Neuroimage Clin. 2022:34:103018). According to a round of cognitive function follow-up in the VICA stroke cohort (N=278), the incidence of cognitive impairment 3-6 months post-stroke is 33.45%. With a two-sided test level α set at 0.05 and a power of the test (1-β) at 0.90, the PASS software was used to calculate the required sample sizes at baseline to be 955 and 3865 individuals, respectively. Considering subsequent attrition and omics data quality control, an additional 20% of the sample size is added to the maximum required, totaling 4638 individuals. The current sample size of 5000 individuals is sufficient for the study needs, with an expected 1200 stroke patients developing PSCI upon completion of the follow-up.

The Complete List of VICA Hospital Centers

Center Name	Center Province
Huashan Hospital, Fudan University	Shanghai
Beijing Tiantan Hospital, Capital Medical University	Beijing
Xuanwu Hospital, Capital Medical University	Beijing
West China Hospital, Sichuan University	Sichuan Province
Zhongnan Hospital of Wuhan University	Hubei Province
The First Affiliated Hospital of Anhui Medical University	Anhui Province
Shanghai Tenth People's Hospital	Shanghai
The Second Affiliated Hospital of Wenzhou Medical University	Zhejiang Province
The First Hospital of Shanxi Medical University	Shanxi Province
The First Affiliated Hospital of Harbin Medical University	Heilongjiang Province
Qingdao Central Hospital	Shandong Province
The Fifth People's Hospital of Shanghai, Fudan University	Shanghai
The First Affiliated Hospital of Kunming Medical University	Yunnan Province
Zhejiang Hospital	Zhejiang Province
The Second Affiliated Hospital of Harbin Medical University	Heilongjiang Province
The First People's Hospital of Hangzhou, Zhejiang University School of Medicine	Zhejiang Province
Zhejiang Provincial People's Hospital	Zhejiang Province
The First Affiliated Hospital of Zhengzhou University	Henan Province
The Affiliated Hospital of Guizhou Medical University	Guizhou Province
School of Public Health, Wuhan University	Hubei Province
Guizhou Hospital of The First Affiliated Hospital of Sun Yat-sen University	Guizhou Province
The Affiliated Hospital of Zunyi Medical University	Guizhou Province
The Fourth People's Hospital of Shanghai, Tongji University	Shanghai
Yongzhou Central Hospital of Hunan Province	Hunan Province
Donggang Central Hospital	Liaoning Province
Wuhan First Hospital	Hubei Province
The Second Hospital of Hebei Medical University	Hebei Province
Benxi Central Hospital	Liaoning Province
Hubei Province Third People's Hospital	Hubei Province

The Affiliated Min Da Hospital of Hubei University for Nationalities	Hubei Province
The First Affiliated Hospital of Guangxi University of Chinese Medicine	Guangxi Zhuang Autonomous Region
The First People's Hospital of Nanning	Guangxi Zhuang Autonomous Region
Shenyang Fifth People's Hospital	Liaoning Province
Xuzhou Central Hospital	Jiangsu Province
The First Affiliated Hospital of Jinzhou Medical University	Liaoning Province
The Affiliated Hongqi Hospital of Mudanjiang Medical College	Heilongjiang Province
Weifang People's Hospital	Shandong Province
The First Hospital of Huai'an Affiliated to Nanjing Medical University	Jiangsu Province
Dongguan People's Hospital	Guangdong Province
Suzhou University Dushu Lake Hospital	Jiangsu Province
Taizhou People's Hospital of Zhejiang Province	Zhejiang Province
The Second Affiliated Hospital of Guizhou University of Chinese Medicine	Guizhou Province
The Affiliated Fuyang Hospital of Anhui Medical University	Anhui Province
Yangpu District Central Hospital of Shanghai	Shanghai
Zhengzhou Central Hospital	Henan Province
Guangdong Provincial Hospital of Chinese Medicine	Guangdong Province
The First Affiliated Hospital of Chengdu Medical College	Sichuan Province
Guiyang Jinyang Hospital, The Second People's Hospital of Guiyang	Guizhou Province
The Third People's Hospital of Bengbu	Anhui Province
Ma'anshan People's Hospital	Anhui Province
The Fifth Affiliated Hospital of Sun Yat-sen University	Guangdong Province
Shiyan People's Hospital	Hubei Province
The Affiliated Hospital of Jining Medical College	Shandong Province
Songjiang District Sijing Hospital of Shanghai	Shanghai
Taikang Tongji Hospital	Hubei Province
Jinshan Branch of Shanghai Sixth People's Hospital	Shanghai
Putuo District People's Hospital of Shanghai	Shanghai
Zhuzhou Central Hospital	Hunan Province
Dong Hospital of Yangpu District	Shanghai

Xinxian People's Hospital	Shandong Province
Tongren People's Hospital	Guizhou Province
Enshi Tujia and Miao Autonomous Prefecture Central Hospital	Hubei Province
Shandong Health Care Group Licheng Central Hospital	Shandong Province
Pudong New Area People's Hospital of Shanghai	Shanghai
Sanshui District People's Hospital of Foshan	Guangdong Province
Shuyang Hospital of Jiangsu Province	Jiangsu Province
Guangdong Qi Fu Hospital	Guangdong Province
The First Affiliated Hospital of Tianjin University of Traditional Chinese Medicine	Tianjin
Macheng People's Hospital	Hubei Province
Guangzhou Red Cross Hospital	Guangdong Province
The Third Hospital of Hebei Medical University	Hebei Province
Linyi People's Hospital	Shandong Province
Ningbo Li Hui Li Medical Center	Zhejiang Province
The Affiliated Hospital of Yangzhou University	Jiangsu Province
China Wuyi Group Hospital	Sichuan Province
Guiyang Sixth Hospital of Guizhou Province	Guizhou Province
Mianyang Third People's Hospital	Sichuan Province
Mianyang Central Hospital	Sichuan Province
The Affiliated Heping Hospital of Changzhi Medical College	Shanxi Province
Dalian University Zhongshan Hospital	Liaoning Province
The Affiliated Hospital of Yan'an University	Shaanxi Province
Sichuan Bayi Rehabilitation Center	Sichuan Province
Wuhu First People's Hospital	Anhui Province
Tai'an Central Hospital	Shandong Province
The Affiliated Hongqi Hospital of Mudanjiang Medical College	Heilongjiang Province
Ordos Central Hospital	Inner Mongolia Autonomous Region
Zhengzhou People's Hospital	Henan Province
Zibo Central Hospital	Shandong Province
The Affiliated Hospital of Southwest Medical University	Sichuan Province
Xuzhou First People's Hospital	Jiangsu Province
Zigong First People's Hospital	Sichuan Province

The First Affiliated Hospital of University of Science and Technology of China	Anhui Province
Shaanxi Provincial People's Hospital	Shaanxi Province
Tianjin Huanhu Hospital	Tianjin
Rizhao People's Hospital	Shandong Province
The First Affiliated Hospital of Jinan University	Guangdong Province
Xianyang Hospital of Yan'an University	Shaanxi Province
Tangshan Central Hospital	Hebei Province
The Second Hospital of Shandong University	Shandong Province
Qinghai Provincial People's Hospital	Qinghai Province
Dandong First Hospital	Liaoning Province
The First Affiliated Hospital of Xinxiang Medical University	Henan Province
Dandong Central Hospital	Liaoning Province
Jinzhou Central Hospital	Liaoning Province
Tianjin People's Hospital	Tianjin
Ningxia Hui Autonomous Region People's Hospital	Ningxia Hui Autonomous Region
The Affiliated Hospital of Guangdong Medical University	Guangdong Province
The Third Hospital of Jilin University	Jilin Province
Liuzhou People's Hospital	Guangxi Zhuang Autonomous Region
Chaoyang Second Hospital	Liaoning Province
Fuxin Central Hospital	Liaoning Province
Nanchong Mental Health Center (Nanchong Second People's Hospital, Nanchong Geriatric Hospital)	Sichuan Province
Suining Central Hospital	Sichuan Province
Huludao Central Hospital	Liaoning Province
Tieling Central Hospital	Liaoning Province
Guizhou Provincial People's Hospital	Guizhou Province
Anshan Central Hospital	Liaoning Province
Dezhou Hospital of Qilu Hospital of Shandong University	Shandong Province
Shanxi Provincial People's Hospital	Shanxi Province
Zhengzhou Ninth People's Hospital	Henan Province
Shijiazhuang Traditional Chinese Medicine Hospital	Hebei Province
Zibo First People's Hospital	Shandong Province
The Affiliated Hospital of Chengdu University of Traditional Chinese Medicine	Sichuan Province
The First People's Hospital of Yunnan Province	Yunnan Province

Jiande First People's Hospital	Zhejiang Province
The First Affiliated Hospital of Guizhou University of Chinese Medicine	Guizhou Province
Panjin Central Hospital	Liaoning Province
Heze Third People's Hospital	Shandong Province
Dali Bai Autonomous Prefecture People's Hospital	Yunnan Province
Liaoning Health Industry Group Fuxin Mining General Hospital	Liaoning Province
The Affiliated Hospital of Xinjiang Medical University	Xinjiang Uyghur Autonomous Region
Wenzhou People's Hospital	Zhejiang Province
Jiangning Hospital of Nanjing	Jiangsu Province
Nanyang Central Hospital	Henan Province
The Second Affiliated Hospital of Guangxi University of Chinese Medicine	Guangxi Zhuang Autonomous Region

Ethic and Informed Consent

Recognizing the potential burden of extensive imaging, neuropsychological assessments, and biological sample collections, particularly for elderly participants, we have implemented measures to protect their rights and minimize impact. A thorough informed consent process is conducted by a physician to ensure participants fully understand the purpose, procedures, potential risks, benefits, and their rights of the study. Participants are compensated appropriately, with waived costs for blood tests and cognitive assessments, and are offered free follow-up consultations. They are also informed of their right to withdraw from the study at any time without consequences to their medical care.

Control Attrition

To ensure the validity and reliability of results in the VICA study, which requires long-term follow-up and extensive data collection, these measures are taken to control the attrition rate.

- Health Education: Enrolled patients receive health education to improve their knowledge of VCI, which is crucial for long-term participation in the VICA study.
- Regular Online Health Follow-ups: Ongoing communication is maintained with participants and their families through regular online health check-ins, which helps in monitoring their condition and reducing attrition.
- On-Site Follow-ups for Mobility Issues: For patients with limited mobility, on-site follow-ups are arranged to ensure they can participate in the study without undue hardship.
- Collaboration with Healthcare Facilities: The study collaborates with community and rehabilitation hospitals to provide comprehensive care and support for patients, which can help in retaining participants.
- Prompt Sharing of Results: Results and reports are shared with participants promptly after follow-ups, which helps in building trust and encouraging continued participation.
- Personalized Advice: Personalized advice is offered to participants based on their follow-up results, which can enhance their engagement with the study.
- Coordination Between Units: Effective coordination between different units involved in the study ensures that patient information is communicated promptly and that timely contact is established, which is essential for maintaining data integrity.

Status of VICA Center Construction

Community Situation of VRF Cohort					
**Neighborhood**	**Age**	**Existing Cohort Size**	**Inclusion number (based on VICA criteria)**	**Inclusion rate (based on VICA criteria)**	**Reference**
**Shanghai, Jing'an Temple**	60+	3141	1337	42.57%	Neuroepidemiology (2014) 43 (2): 114–122.
**Taizhou, Hutou, Lubao and Caixiang**	55-65	904	300	33.19%	Alzheimers Dement. 2021 Jan;17(1):18-28.
**Shanghai, Hongmei Road**	40-80	750	228	24.00%	
**Wuhan, Fruit Lake**	40-80	514	180	35.00%	

Center construction of CSVD cohort					
	**Target Center Count**	**Existing Center Count**	**Planned Enrollment Number**	**Monthly CSVD Outpatient Visits at Existing Centers**	**Monthly CSVD Enrollments at Existing Centers**
**First-tier**	60	30	1500	3000+	300+
**Second-tier**	30	15	1000	1500+	80+
**Third-tier**	10	5	500	700+	30+
**Sum**	100	50	3000	5300+	400+

Center construction of STROKE cohort					
	**Target Center Count**	**Existing Center Count**	**Planned Enrollment Number**	**Monthly Hospitalized Stroke Patients at Existing Sites**	**Monthly STROKE Enrollments at Existing Sites**
**First-tier**	60	57	3000	3400+	600+
**Second-tier**	30	28	1500	2700+	270+
**Third-tier**	10	5	500	1000+	50+
**Sum**	100	90	5000	7100+	900+
